# Turn-on Luminescent Probe for Hydrogen Peroxide Sensing and Imaging in Living Cells based on an Iridium(III) Complex–Silver Nanoparticle Platform

**DOI:** 10.1038/s41598-017-09478-6

**Published:** 2017-08-21

**Authors:** Jinshui Liu, Zhen-Zhen Dong, Chao Yang, Guodong Li, Chun Wu, Fu-Wa Lee, Chung-Hang Leung, Dik-Lung Ma

**Affiliations:** 1 College of Chemistry and Materials Science, Anhui Normal University, Wuhu, China; 2 Department of Chemistry, Hong Kong Baptist University, Kowloon Tong, Hong Kong China; 3 State Key Laboratory of Quality Research in Chinese Medicine, Institute of Chinese Medical Sciences, University of Macau, Macao, China; 4 College of International Education, School of Continuing Education, Hong Kong Baptist University, Shek Mun, Hong Kong China

## Abstract

A sensitive turn-on luminescent sensor for H_2_O_2_ based on the silver nanoparticle (AgNP)-mediated quenching of an luminescent Ir(III) complex (**Ir-1**) has been designed. In the absence of H_2_O_2_, the luminescence intensity of **Ir-1** can be quenched by AgNPs via non-radiative energy transfer. However, H_2_O_2_ can oxidize AgNPs to soluble Ag^+^ cations, which restores the luminescence of **Ir-1**. The sensing platform displayed a sensitive response to H_2_O_2_ in the range of 0−17 μM, with a detection limit of 0.3 μM. Importantly, the probe was successfully applied to monitor intracellular H_2_O_2_ in living cells, and it also showed high selectivity for H_2_O_2_ over other interfering substances.

## Introduction

H_2_O_2_ is widely used in industry and households for rinsing, bleaching and disinfection. For example, in the food industry, H_2_O_2_ is used to replace chlorine-containing bleaching and sterilizing agents^1^. It also plays an important role in many biological processes and enzymatic reactions, particularly those related to intracellular oxidative stress^2^. In fact, escalated levels of H_2_O_2_ can cause irreversible cellular damage through the oxidation of biomolecules, leading to cell death^3^. Moreover, oxidative damage to cellular proteins, nucleic acids, and lipid molecules are associated with aging and age-related disorder ranging from neurodegeneration to diabetes^3, 4^. Therefore, a rapid and reliable detection of H_2_O_2_ is important in pharmaceutical, clinical, and food industries.

Multiple methods such as spectrophotometry^5, 6^, chemiluminescence^7^ and electrocatalysis^8^ have been developed for the detection of H_2_O_2_. Specifically, biosensors have been developed on the basis of electrocatalysis of immobilized enzymes arising from H_2_O_2_ reduction^9^. However, the enzyme-based biosensors are limited by sensitivity to environmental conditions, high cost, short shelf-life and complicated immobilization procedures^10–12^. Meanwhile, fluorescent strategies have lots of advantages, particularly rapid response, high sensitivity, and simple manipulation^13, 14^. Various fluorescence probes such as organic molecules^15^, carbon dots^16, 17^, metal nanoclusters^18^, and nanoparticles^19–21^, have good performance on the determination of H_2_O_2_. However, there are still some drawbacks for these reported probes, including poor sensitivity and selectivity, low stability in biological environment, or complicated operation^17, 18, 22^. Fluorescence turn-on sensors are generally more desirable than fluorescence quenching sensors as the former is less susceptible to false positive signals^23, 24^.

Luminescent Ir(III) complexes have been employed to detect a variety of analytes^25–27^. Compared with organic molecules, Ir(III) complexes generally exhibit large Stokes shifts, ease in synthesis and long-lived luminescence which could be distinguished from fluorescence noise in biological matrices^26, 27^. Meanwhile, silver nanoparticles (AgNPs) form a promising nanomaterial that has been developed in many applications because of their remarkable properties, such as high extinction coefficient and surface plasmon resonance absorption^28–30^. It has been reported that AgNPs can be oxidized by traces of H_2_O_2_, to form Ag^+^^31^_._ In addition, AgNPs can function as excellent quenchers for fluorescent materials, such as organic dyes and quantum dots (QDs)^32–35^. However, as far as we know, the application of the Ir(III) complexes combined with AgNPs has not yet been reported in the literature for H_2_O_2_ sensing. Consequently, taking advantages of the Ir(III) complex (**Ir-1**, [Ir(tfppy)_2_(pyphen)]^+^, where tfppy = 2-[4-(trifluoromethyl)phenyl]pyridine, pyphen = pyrazino[2,3-*f*][1,10]phenanthroline) and AgNPs, we designed a novel turn-on luminescent probe for rapid and sensitive detection of intracellular H_2_O_2_.

**Figure 1:**
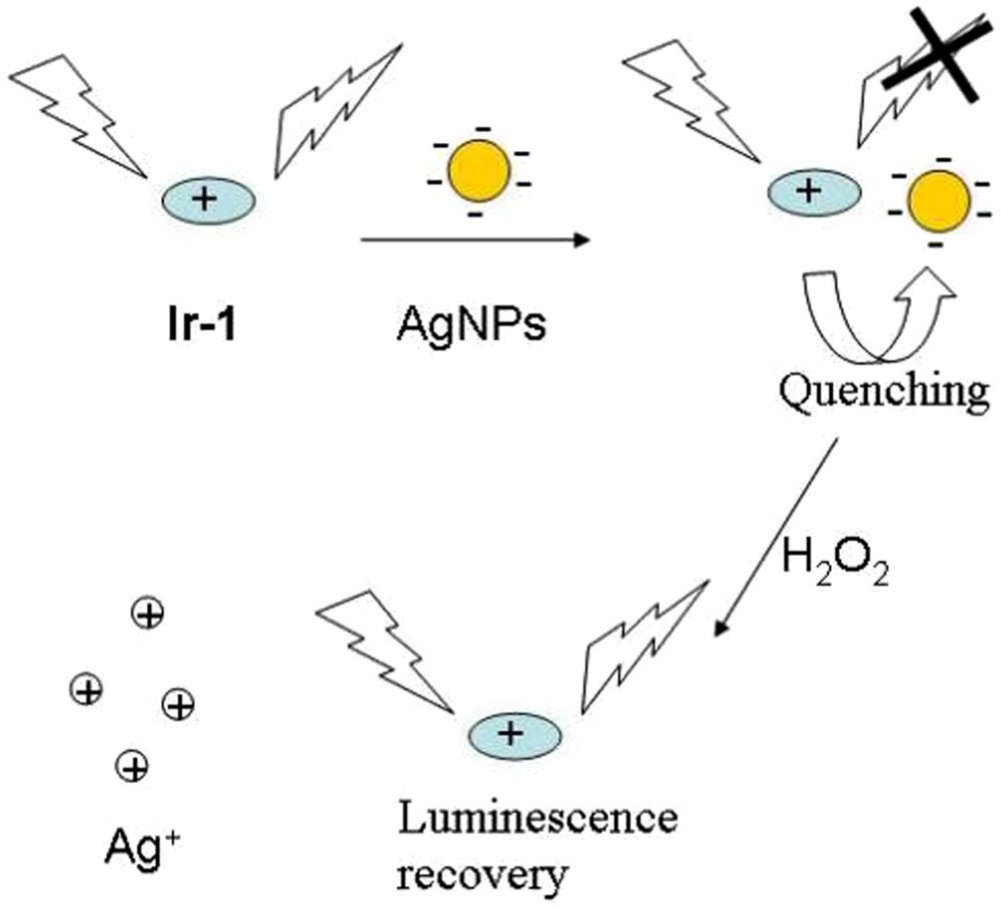
Illustration of the design rationale for the detection of H_2_O_2_ using a luminescence sensor based on **Ir-1**–AgNPs system.

The sensing mechanism of the **Ir-1**–AgNP probe for H_2_O_2_ is illustrated in Fig. 1. In the initial system, the luminescence of **Ir-1** was significantly quenched by AgNPs. However, this AgNPs-induced quenching effect can be reversed by H_2_O_2_ due to oxidation of AgNPs to Ag^+^. To our knowledge, the **Ir-1**–AgNP is the first application of the combination of Ir(III) complexes and AgNPs for H_2_O_2_ sensing in both aqueous solutions and living cells.

## Results and Discussion

### Sensing Mechanism

**Figure 2:**
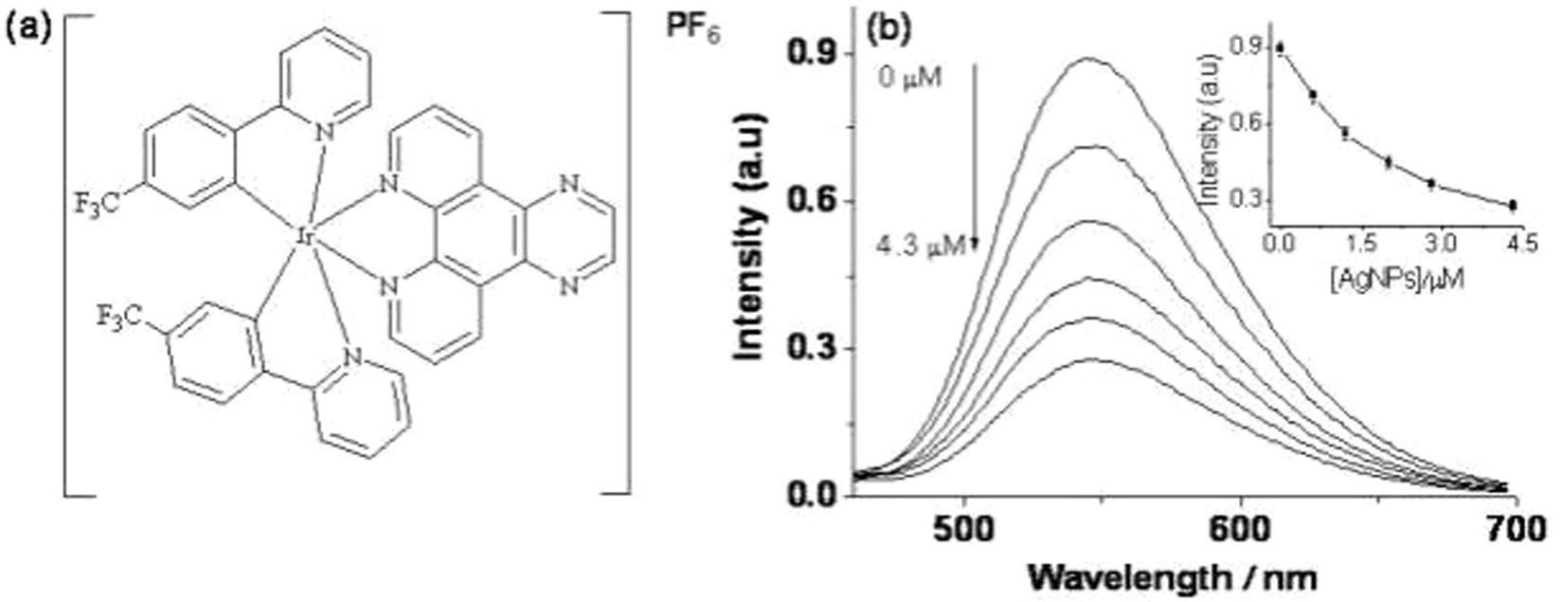
(**a**) Chemical structure of **Ir-1**. (**b**) Luminescence emission spectra 0.3 μM **Ir-1** in Tris-HNO_3_ buffer solution (pH 7.0) containing different concentrations of AgNPs. The inset is the luminescence intensity at 545 nm plotted against the AgNPs concentration.

**Ir-1**, carrying tfppy as its C^N ligand and pyphen as its N^N ligand (Fig. 2a), was characterized by^1^H-NMR,^13^C-NMR and HRMS (Figs S1–S3 and Table S1). **Ir-1** emits strong luminescence at 545 nm under the excitation of 295 nm in aqueous buffer solution. As expected, the luminescence of **Ir-1** decreased gradually with increasing amounts of AgNPs in solution (Fig. 2b). This is because the positively charged **Ir-1** could be adsorbed on the surface of the citrate-stabilized AgNPs through electrostatic interactions, which efficiently quenched the luminescence of **Ir-1**. However, the luminescence could be recovered in the presence of H_2_O_2_ attributed to oxidation of AgNPs into soluble Ag^+^ by H_2_O_2_. In order to study the kinetic behavior between the **Ir-1**–AgNP system and H_2_O_2_, the luminescence change was monitored as a function of time. As shown in Fig. S4, the luminescence intensity of the **Ir-1**–AgNP system increased with time and reached the plateau after 10 min, indicating that the reaction between AgNPs and H_2_O_2_ at ambient temperature is rapid. In the absence of AgNPs, H_2_O_2_ showed no apparent effect on the luminescence of **Ir-1** (Fig. S5). Therefore, the increase in the luminescence of the system should arise primarily from the decomposition of AgNPs by H_2_O_2_, which restores the emission of **Ir-1**.

**Figure 3:**
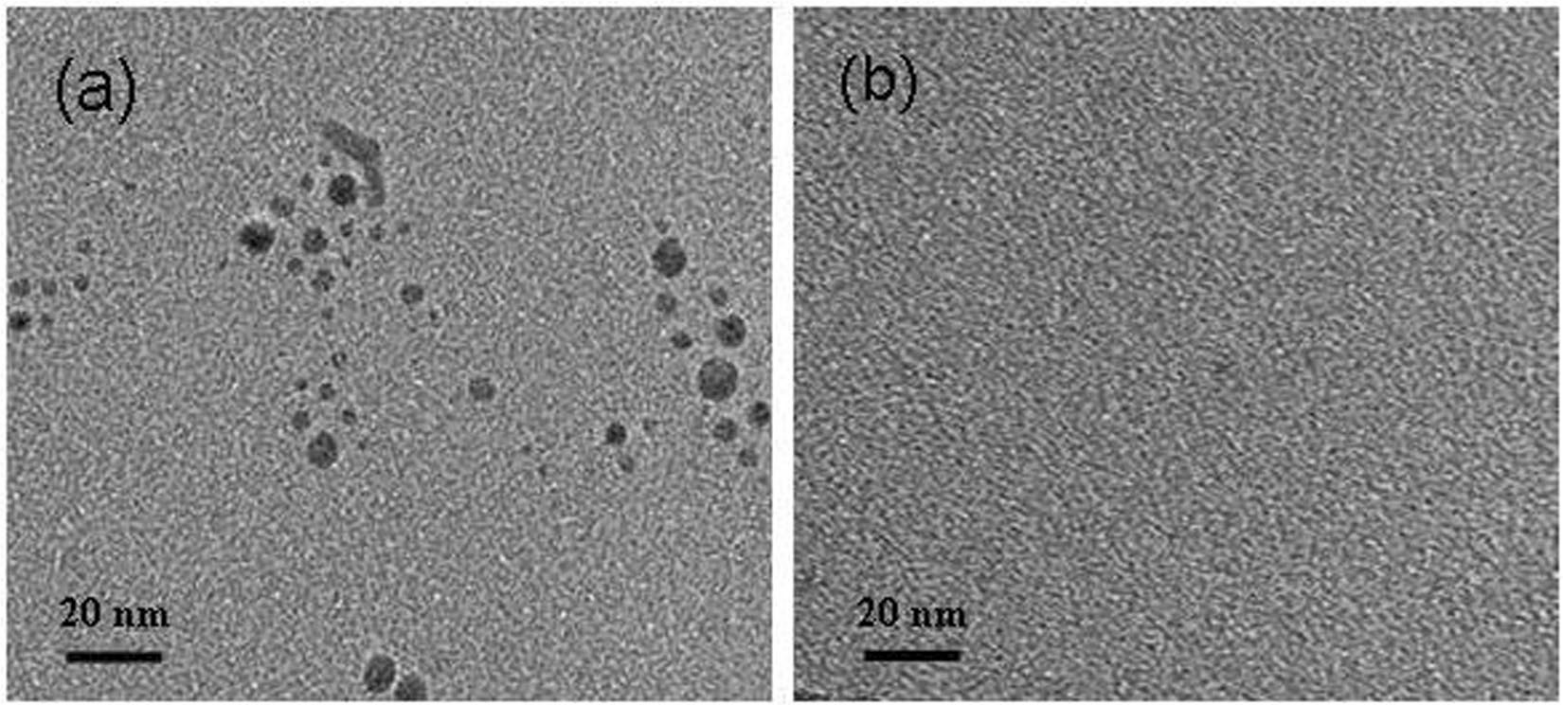
Transmission electron microscopy images of (**a**) AgNPs and (**b**) AgNPs in the presence of H_2_O_2_.

The mechanism involved in the luminescence quenching and recovery process was also demonstrated by transmission electron microscopy (TEM) imaging. In the absence of the **Ir-1**, the AgNPs were well-dispersed (Fig. 3a). However, after the addition of **Ir-1**, slight aggregation of AgNPs was observed, suggesting that **Ir-1** and AgNPs interacted on the surface of AgNPs (Fig. S6b). The identity of the **Ir-1**–AgNP complex was further confirmed by energy dispersive X-ray spectroscopy (EDX), which showed strong elemental signals for both Ir and Ag (Fig. S6c). Strikingly, after treatment of AgNPs with H_2_O_2_, no AgNPs could be observed in the TEM images (Fig. 3b). This suggests that the AgNPs were decomposed and transformed to Ag^+^, which is consistent with previously reported^36–38^. The UV–vis absorbance spectra of AgNPs in the absence and presence of H_2_O_2_ are shown in Fig. S7. AgNPs alone showed a strong characteristic surface plasmon resonance peak at around 390 nm^39^. However, the absorption band of AgNPs gradually decreased upon increasing concentration of H_2_O_2_. These phenomena were ascribed to the oxidation of AgNPs to Ag^+^ by H_2_O_2_, leading the decomposition of the AgNPs.

### Sensitivity

**Figure 4:**
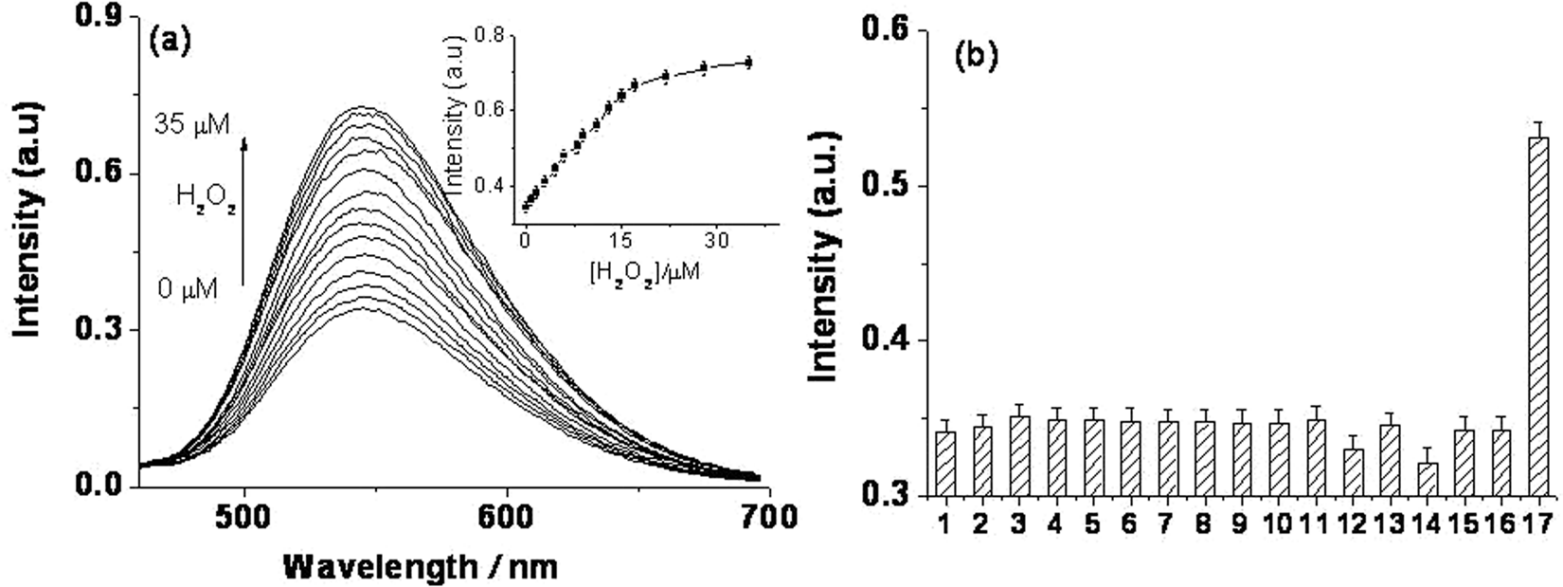
(**a**) Luminescence emission spectra of 1 μM **Ir-1** in Tris-HNO_3_ buffer solution (pH 7.0) containing 2.8 μM AgNPs and different concentrations of H_2_O_2_ (0 μM to 35 μM). The inset is the luminescence intensity plotted against the H_2_O_2_ concentration. (**b**) Luminescence intensity of the **Ir-1**-AgNP system (0.3 μM **Ir-1** and 2.8 μM AgNPs in Tris-HNO_3_ at pH 7.0) in the presence of interfering species (HSA and BSA 50 μg/L, other interfering species 50 μM) or H_2_O_2_ (9 μM) (from 1 to 17: blank, threonine, serine, glycine, ascorbic acid, HSA, BSA, Zn^2+^, Co^2+^, Ni^2+^, Cd^2+^, Fe^3+^, Mg^2+^, Cu^2+^, K^+^, Na^+^, and H_2_O_2_).

To explore the applicability of the proposed luminescence sensor for H_2_O_2_ detection, we studied the luminescence response of the **Ir-1**–AgNP system toward varying concentrations of H_2_O_2_. The luminescence intensity of the system was gradually restored with increasing concentration of H_2_O_2_ (Fig. 4a). Meanwhile, a good linear relationship over the range from 0 to 17 μmol L^−1^ with a correlation coefficient of 0.998 was obtained (Fig. S8). The limit of detection (LOD) was calculated to 0.3 μM according to the signal-to-noise method (S/N = 3). The sensitivity of this method is comparable to other reported methods for H_2_O_2_ detection as summarized in Table S2 ^4, 10–16, 20, 36, 40–44^.

### Selectivity

To assess the selectivity of **Ir-1**–AgNPs system for H_2_O_2_, the influences of metal ions and amino acids were studied. As shown in Fig. 4b, nearly no luminescence changes could be observed with the other substances (Fig. 4b), which demonstrates that the **Ir-1**–AgNP system is highly selective for H_2_O_2_ over other non-target substances.

### Cell imaging

Given the promising capability of **Ir-1** for sensing H_2_O_2_ in aqueous solution, we then investigated the ability of **Ir-1** for monitoring H_2_O_2_ in living human cells. **Ir-1** showed cytotoxicity against HeLa (human cervical cancer) cells with an IC_50_ value of 5.12 μM (Fig. S9).

**Figure 5:**
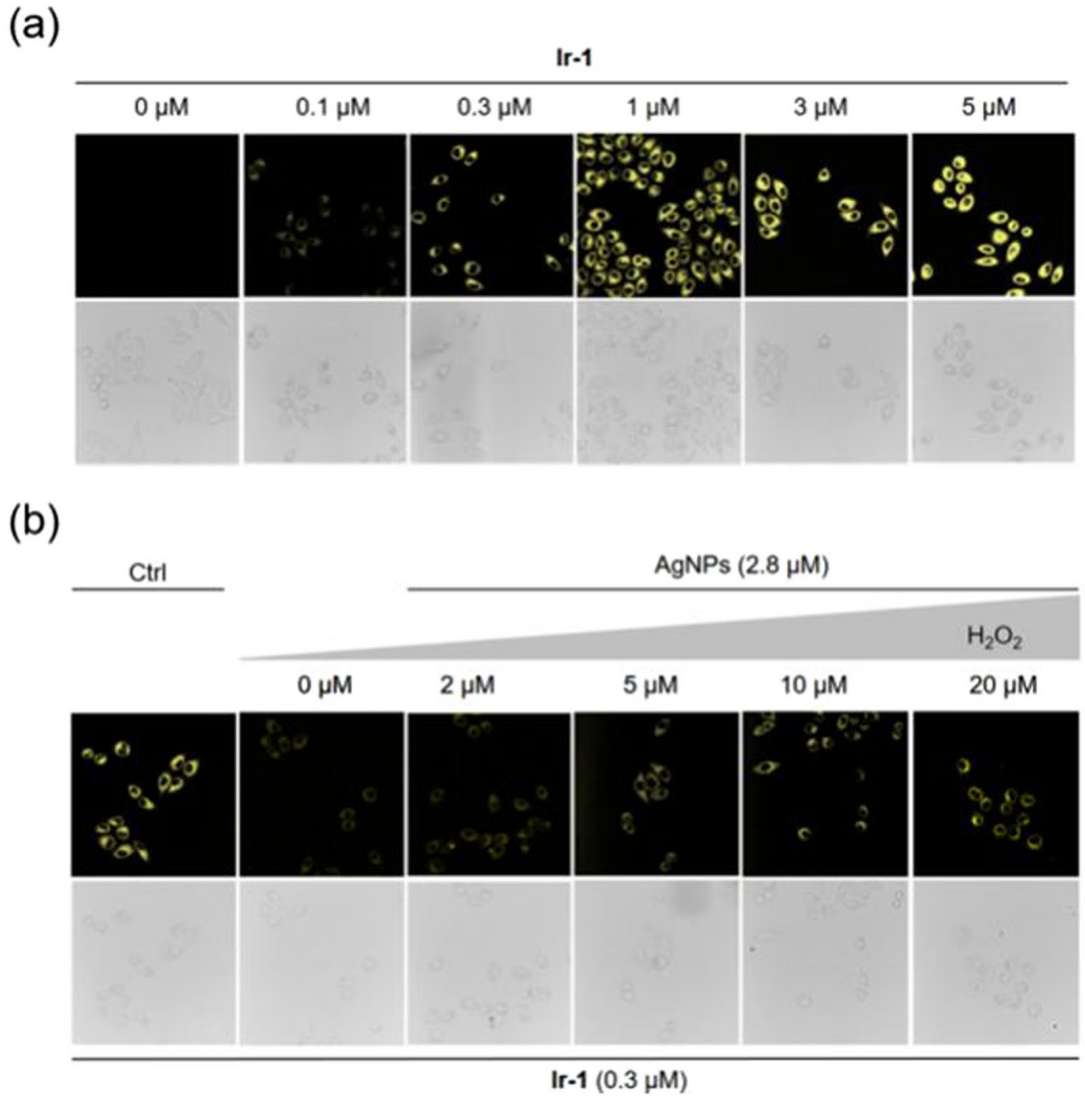
Confocal luminescence microscopy imaging of HeLa cells. (**a**) HeLa cells were incubated with the indicated concentration of **Ir-1** for 1 h. (**b**) HeLa cells were pretreated with **Ir-1** (0.3 μM) and AgNPs (2.8 μM) for 1 h before incubation with different concentration of H_2_O_2_. The upper row is luminescence imaging, and the lower row is bright field imaging. Excitation wavelength = 405 nm.

In the cell imaging study, the luminescence intensity of HeLa cells was enhanced with increasing concentration of **Ir-1** (Fig. 5a), showing that **Ir-1** could effectively penetrate into cells. A concentration of 0.3 μM of **Ir-1** was chosen for subsequent cell experiments as this concentration was over 10-fold lower than the IC_50_ value for cytotoxicity, while it still gave a good luminescence signal.

Next, HeLa cells were pretreated with **Ir-1** (0.3 μM) for 1 h before incubation with different concentration of AgNPs. The luminescence intensity of HeLa cells was remarkably reduced with increasing concentration of AgNPs (Fig. S10), which was attributed to AgNPs-mediated quenching of an luminescent **Ir-1** as described previously. However, when H_2_O_2_ was added into the growth medium for another 1 h, the luminescence of HeLa cells was recovered in a dose-dependent manner (Fig. 5b). Collectively, these results suggest that **Ir-1**–AgNP can be developed for the monitoring of H_2_O_2_ levels in living cells.

## Conclusion

Consequently, we have proposed a turn-on luminescence assay for H_2_O_2_ detection employing the **Ir-1**–AgNP system. In this nano-composite system, **Ir-1** functioned as a luminescence reporter, while AgNPs were employed both as a luminescence quencher and as a recognition unit for H_2_O_2_. Based on the luminescence recovery of the **Ir-1**–AgNP system triggered by H_2_O_2_, this nanoprobe was successfully applied to detect H_2_O_2_ at the intracellular level in living cells. In addition, the **Ir-1**–AgNP probe possesses some superior properties, including label-free, good sensitivity and selectivity, low cost, easy manipulation, low cytotoxicity, and turn-on luminescent response. To our knowledge, the probe is the first combination of Ir(III) and AgNPs applied for the detection of H_2_O_2_ in living cells reported in the literature.

## Materials and Methods

### Chemicals and materials

Iridium chloride hydrate (IrCl_3_·xH_2_O) was purchased from Precious Metals Online (Australia). Other reagents were purchased from Sigma Aldrich (St. Louis, MO) and used as received. All of the reagents were of analytical grade and were used as received without further purification. All solutions were prepared in Milli-Q water under ambient conditions. HeLa cell lines were obtained from ATCC (Manassas, VA, USA). Dulbecco’s Modified Eagle’s medium, fetal bovine serum, penicillin and streptomycin were obtained from Sigma-Aldrich Co. LLC (St. Louis, MO, USA).

### Synthesis of AgNPs

AgNPs were fabricated according to reported methods with slight modifications^28, 45^. In a typical procedure, 0.08 mL AgNO_3_ (0.1 M) and 0.1 mL trisodium citrate (0.1 M) were mixed into 100 mL pure water and stirred under the condition of ice bath. Then, freshly prepared NaBH_4_ solution was added dropwise into the mixture until it turned yellow. The resulting yellow solution was stirred for another 30 min to form AgNPs quantitatively, which was stored at 4 °C for subsequent use. The diameter of AgNPs prepared was measured to be 8–9 nm by transmission electron microscopy (TEM).

### Synthesis of Ir-1

**Ir-1** was synthesized based on a reported literature method^46–49^. [Ir_2_(tfppy)_4_Cl_2_] (0.2 mmol) and pyppy (0.42 mmol) in a mixed solvent of DCM:methanol (1:1.2 (v/v), 36 mL) was refluxed overnight. The reaction mixture was allowed to cool to ambient temperature, and unreacted cyclometallated dimer was removed by filtration. Excess ammonium hexafluorophosphate was then added into the filtrate, and the resulting mixture was stirred for another 30 min. Afterwards, the solution was evaporated under reduced pressure until precipitation was initiated. The precipitate was filtered, and washed by several portions of water and diethyl ether. The crude product was then recrystallized by the acetonitrile/diethyl ether vapor diffusion to obtain the desired compound, which was characterized by^1^H-NMR,^13^C-NMR, high resolution mass spectrometry (HRMS) and elemental analysis.

### Luminescence response of Ir-1 towards AgNPs

**Ir-1** (0.3 μM) was added to varying concentrations of AgNPs in Tris-HNO_3_ buffer (5 mM Tris-HNO_3_, pH 7.0), then their emission intensity were measured.

### Detection of H_2_O_2_

A series of sample solutions of same composition was prepared by mixing **Ir-1** (0.3 μM) with AgNPs (2.8 μM) in Tris-HNO_3_ buffer (5 mM Tris-HNO_3_, pH 7.0). Upon individual addition of varying concentrations of stock H_2_O_2_ solution, the sample solutions were incubated for 10 min at room temperature. Emission spectra were collected in the range of 450–700 nm at the excitation wavelength of 295 nm.

### Cell imaging

HeLa cells were pretreated with **Ir-1** (0.3 μM) for 1 h at 37 °C, then AgNPs of different concentrations (0 μM, 0.1 μM, 0.3 μM, 1 μM, 3 μM and 5 μM) was added before further incubation for 1 h. After washing with PBS three times, the luminescence intensity of HeLa cells was imaged by a Leica SP8 laser scanning confocal microscope upon excitation at 405 nm.

For H_2_O_2_ detection, the experiment was performed as above except that after incubation in the presence of AgNPs (2.8 μM), cells were further treated with H_2_O_2_ ranging from 0 to 20 μM for 1 h. After washing with PBS three times, the luminescence intensity of HeLa cells was then imaged as above.

### Statistics analysis

One-way analysis of variance (ANOVA) followed by the Dunnett’s method for multiple comparisons by using GraphPad Prism 6.0 was used to analyse the data.

## Electronic supplementary material

Supplementary Info File #1
