# Supplementary material for: Turn-on Luminescent Probe for Hydrogen Peroxide Sensing and Imaging in Living Cells based on an Iridium(III) Complex–Silver Nanoparticle Platform
Source: Sci Rep. 2017 Aug 21;7:8980. doi: 10.1038/s41598-017-09478-6 (PMC5566206; doi:10.1038/s41598-017-09478-6)
Supplement: Supplementary file 1 — Supplementary Info File #1 [file 41598_2017_9478_MOESM1_ESM.pdf]

Electronic Supporting Information

Turn-on Luminescent Probe for Hydrogen Peroxide Sensing and Imaging in Living Cells based on an Iridium(III) Complex−Silver Nanoparticle Platform

Jinshui Liu[1, 2†], Zhen-Zhen Dong[2†], Chao Yang[3], Guodong Li[3], Chun Wu[2], Fu-Wa Lee[4], Chung-Hang Leung[3] and Dik-Lung Ma[2*]

[1] College of Chemistry and Materials Science, Anhui Normal University, Wuhu, China.

[2] Department of Chemistry, Hong Kong Baptist University, Kowloon Tong, Hong Kong, China.

[3] State Key Laboratory of Quality Research in Chinese Medicine, Institute of Chinese Medical Sciences, University of Macau, Macao, China.

[4] College of International Education, School of Continuing Education, Hong Kong Baptist University, Shek Mun, Hong Kong, China.

[†]These authors contributed equally to this work.

* Corresponding author:

Dr. Dik-Lung Ma, E-mail: edmondma@hkbu.edu.hk, Tel: (+852) 3411-7075, Fax: (+852) 3411-7348.

General experimental. Deuterated solvents for NMR purposes were obtained from Armar and used as received. ^{1}H and ^{13}C NMR were recorded on a Bruker Avance 400 spectrometer operating at 400 MHz (^{1}H) and 100 MHz (^{13}C). ^{1}H and ^{13}C chemical shifts were referenced internally to solvent shift (acetone-*d*6: ^{1}H, 2.05, ^{13}C, 29.8). Chemical shifts (are quoted in ppm, the downfield direction being defined as positive. Uncertainties in chemical shifts are typically ±0.01 ppm for ^{1}H and ±0.05 for ^{13}C. Coupling constants are typically ±0.1 Hz for ^{1}H-^{1}H and ±0.5 Hz for ^{1}H-^{13}C couplings. The following abbreviations are used for convenience in reporting the multiplicity of NMR resonances: s, singlet; d, doublet; t, triplet; q, quartet; m, multiplet; br, broad. All NMR data was acquired and processed using standard Bruker software (Topspin). Mass spectrometry was performed at the Mass Spectroscopy Unit at the Department of Chemistry, Hong Kong Baptist University, Hong Kong (China). HeLa cells were maintained in Dulbecco's Modified Eagle's medium supplemented with 10% fetal bovine serum, 100 U/mL penicillin, and 100 μg/mL streptomycin.

Transmission electron microscopy (TEM). The morphology of the AgNPs was investigated using transmission electron microscopy (TEM, Tecnai G2 20 S-TWIN Transmission Electron Microscope). For TEM measurements, the sample solutions were deposited on an Agar holey carbon-coated copper grid (300 mesh) and dried in a vacuum at room temperature before observation.

Photophysical measurement. Emission spectra and lifetime measurements for complexes were performed on a PTI TimeMaster C720 Spectrometer (Nitrogen laser: pulse output 365 nm). Error limits were estimated: λ (±1 nm); τ (±10%); φ (±10%). UV/Vis absorption spectra were recorded on a Cary UV-300 spectrophotometer (double beam).

Luminescence quantum yields were determined using the method of Demas and Crosby [1] $[Ru(bpy)_3][PF_6]_2$ in degassed acetonitrile as a standard reference solution (Φ_r = 0.062) and calculated according to the reported equation:

$$\Phi_s = \Phi_r(B_r/B_s)(n_s/n_r)^2(D_s/D_r)$$

where the subscripts s and r refer to sample and reference standard solution respectively, n is the refractive index of the solvents, D is the integrated intensity, and Φ is the luminescence quantum yield. The quantity B was calculated by $B = 1 - 10^{-AL}$, where A is the absorbance at the excitation wavelength and L is the optical path length.

Cytotoxicity experiment

HeLa cells were seeded at a density of 8,000 cells per well in 96-well plates and until they reached 70–80% confluence. The medium was then replaced with serum-free medium. **Ir-1** dissolved in DMSO was added to cells at final concentrations ranging from 10 nM to 100 μM under normoxic conditions for 48 h. MTT (3-(4,5-Dimethylthiazol-2-yl)-2,5-Diphenyltetrazolium Bromide) was added to each well at a final concentration 0.5 mg/mL, and then cells were incubated for a further 4 h. The medium was removed from the cells, then 100 μL DMSO was added per well. The plate was shaken for 10 min at room temperature in the dark. The absorbance of the wells was recorded at 490 nm using a SpectraMax M5 microplate reader

Table S1. Photophysical properties of **Ir-1** in acetonitrile at 298 K.

Complex	Quantum yield	λ_{em} / nm	Lifetime / μs	UV/vis absorption<br>λ_{abs} / nm (ε / dm^3 mol^{-1} cm^{-1})
**1**	0.48	545	4.217	257 (9.5 × 10^4), 338 (1.2 × 10^4)

Ir-1. Yield: 68%, ^{1}H NMR (400 MHz, acetone-d_6) δ 9.80 (2H), 9.35 (2H), 8.61 (2H), 8.45 (2H), 8.17-8.25 (4H), 7.95-8.07 (4H), 7,44 (2H), 7.15 (2H), 6.65 (2H); ^{13}C NMR (100 MHz, acetone-d_6) δ 166.86, 153.75, 151.72, 150.25, 149.61, 149.33, 147.89, 140.70, 140.44, 136.48, 131.79, 131.46, 129.28, 128.25, 128.21, 126.42, 126.17, 126.03, 123.72, 122.20, 120.91, 120.87; MALDI-TOFHRMS: Calcd. for $C_{38}H_{22}F_6IrN_6$ [M–PF$_6$]$^+$: 869.14 Found: 869.1411. Anal.: ($C_{38}H_{22}F_{12}IrN_6P.H_2O$) C, H, N: calcd. 44.23, 2.34, 8.14; found 44.54, 2.23, 8.20.

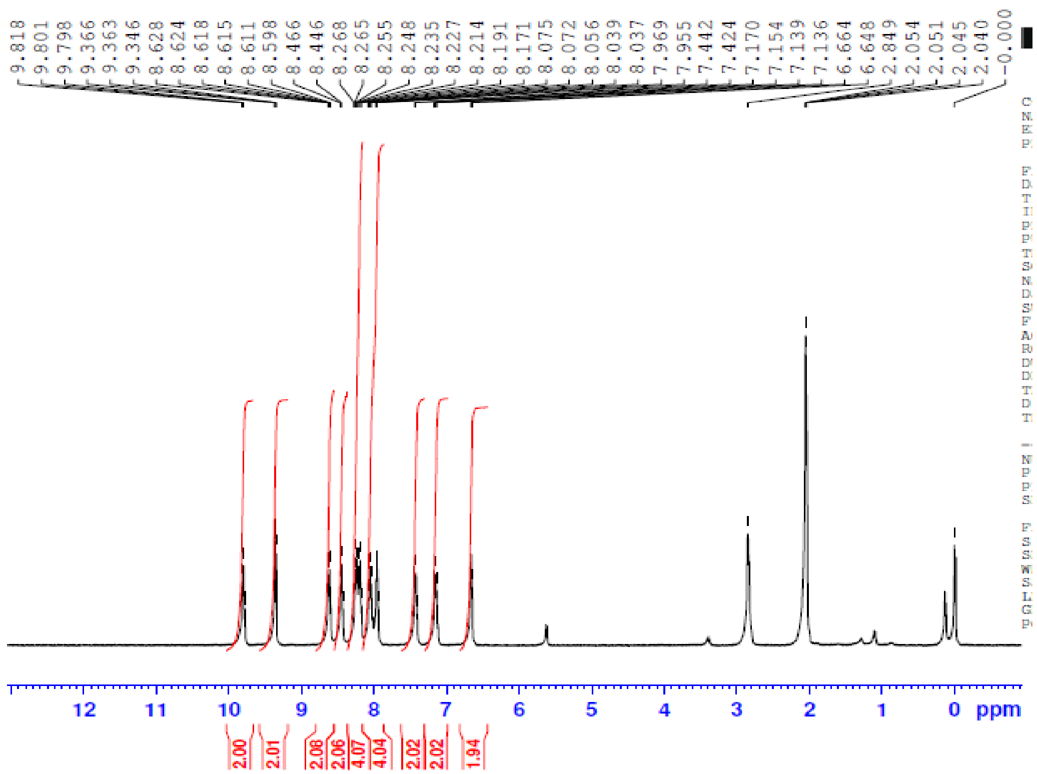

Fig. S1 ^{1}H NMR spectra of **Ir-1**.

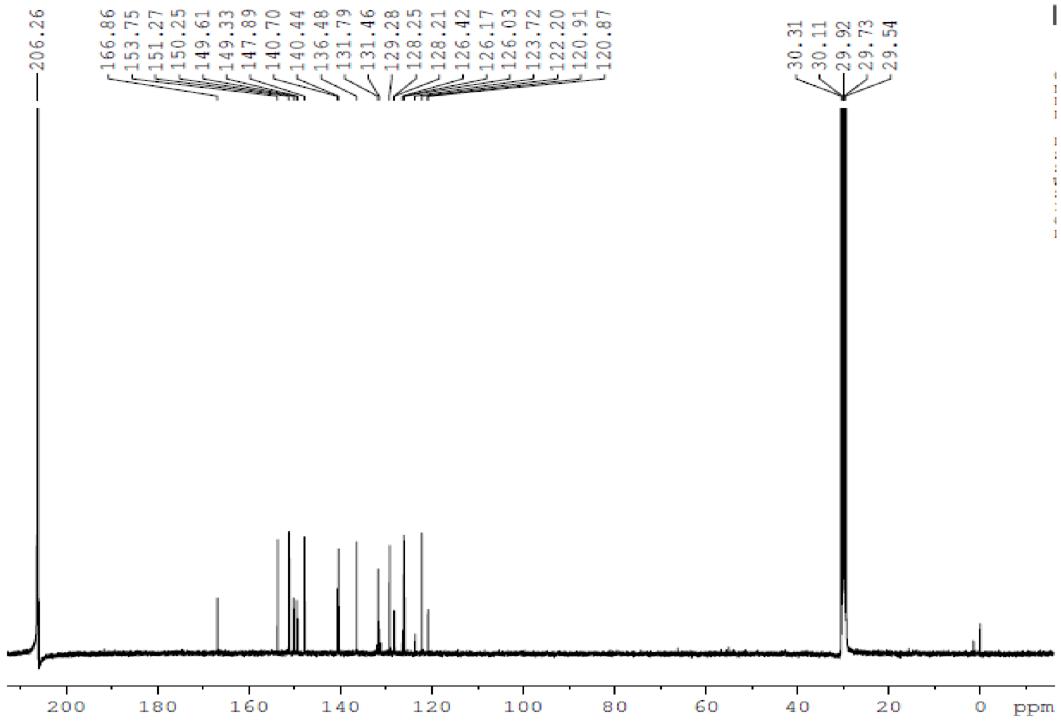

Fig. S2 ^{13}C NMR spectra of **Ir-1**.

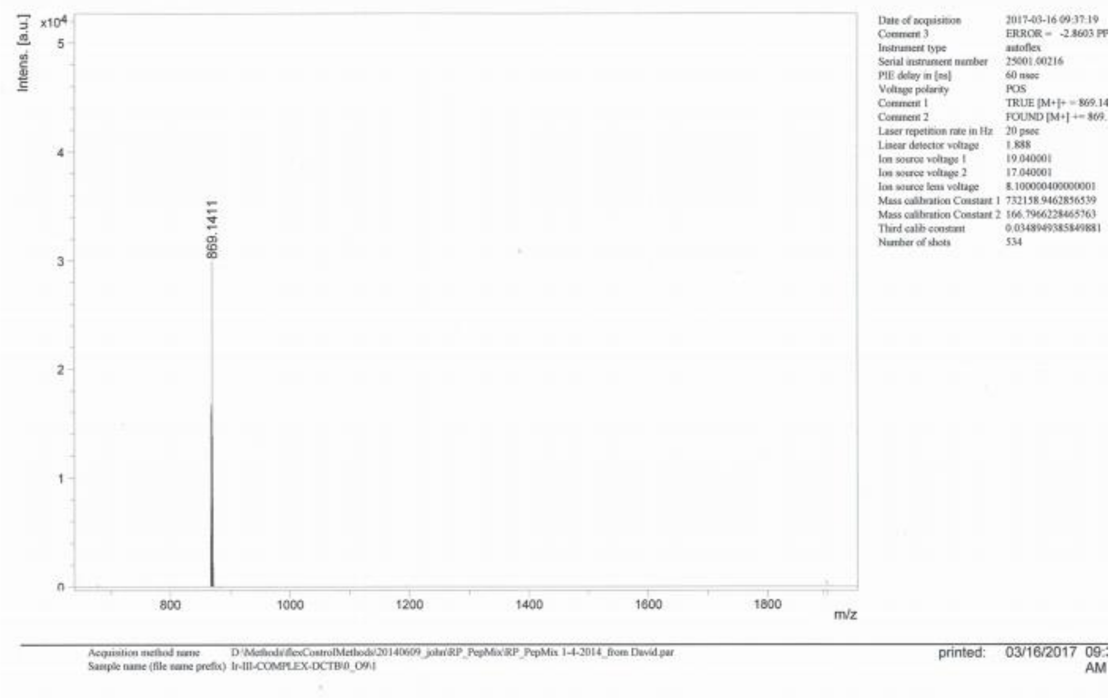

Fig. S3 MALDI-TOF HRMS spectra of **Ir-1**.

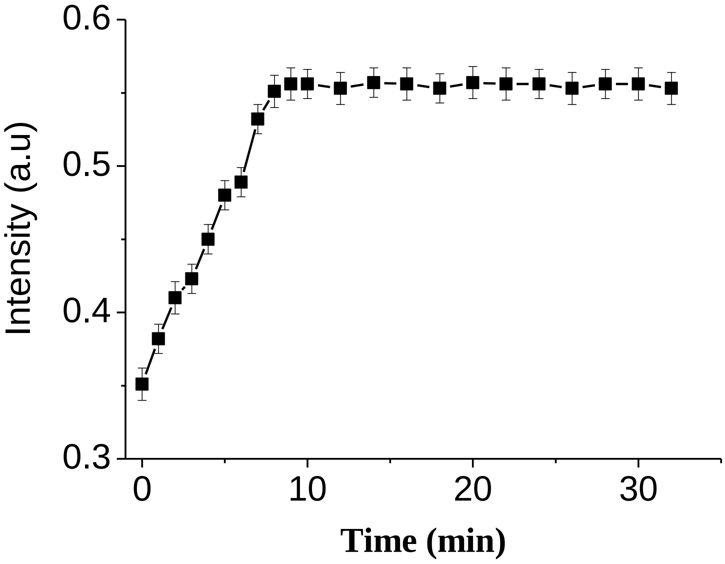

Fig. S4 Luminescence recovery from the **Ir-1**–AgNP system by 9 μM H_2O_2 as a function of time.

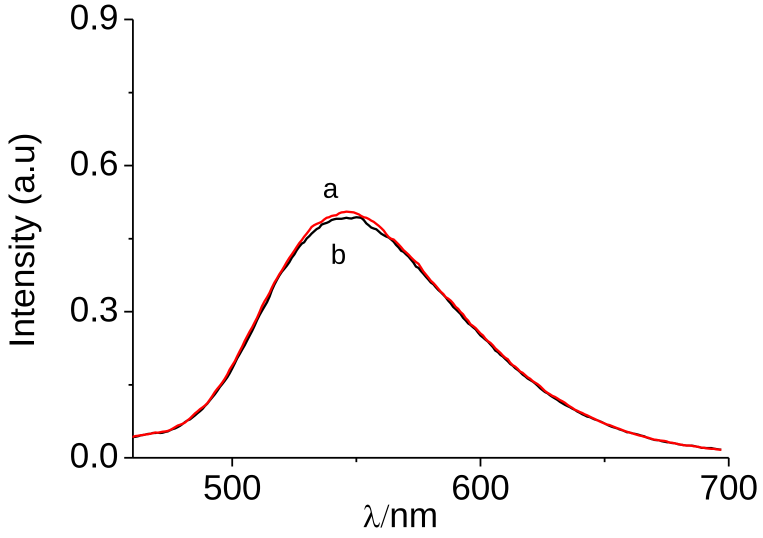

Fig. S5 Luminescence of **Ir-1** in the absence (a) and presence (b) of 9 μM H_2O_2.

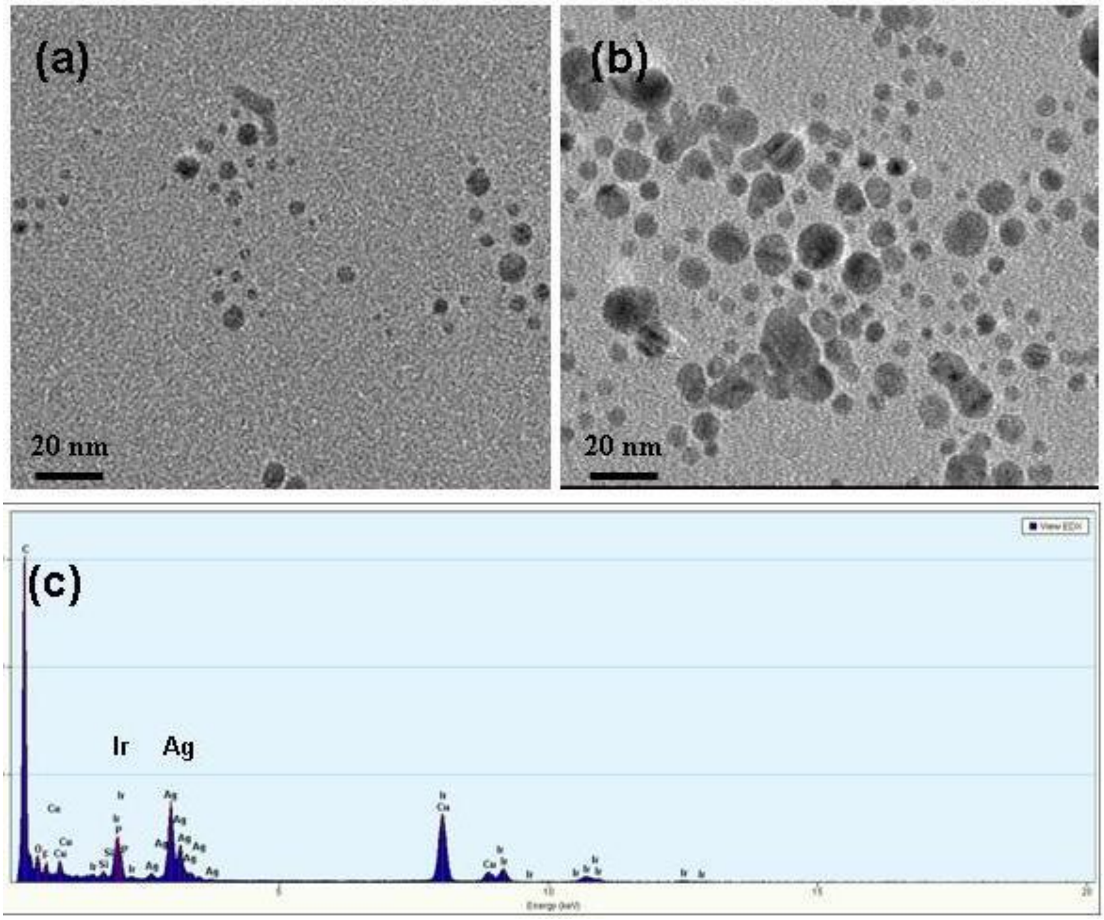

Fig. S6 TEM images of the AgNPs in the (a) absence and (b) presence of **Ir-1**. (c) Energy dispersive X-ray spectrum of the **Ir-1**–AgNP complex

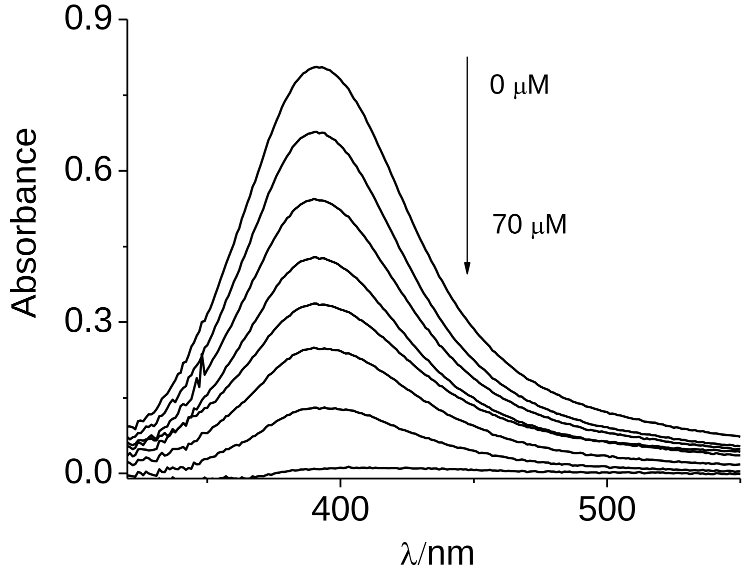

Fig. S7 UV−vis absorption spectra of AgNPs (12 μM) after the addition of different concentrations of H_2O_2 (from the top to the bottom (μM): 0, 10, 20, 30, 40, 50, 60, and 70).

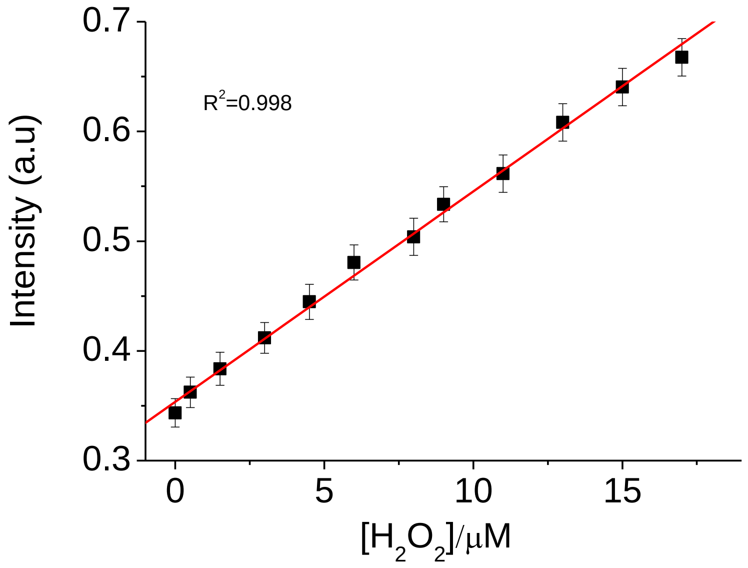

Fig. S8 Linear calibration plots of concentration of H_2O_2 vs. luminescence intensity of **Ir-1**.

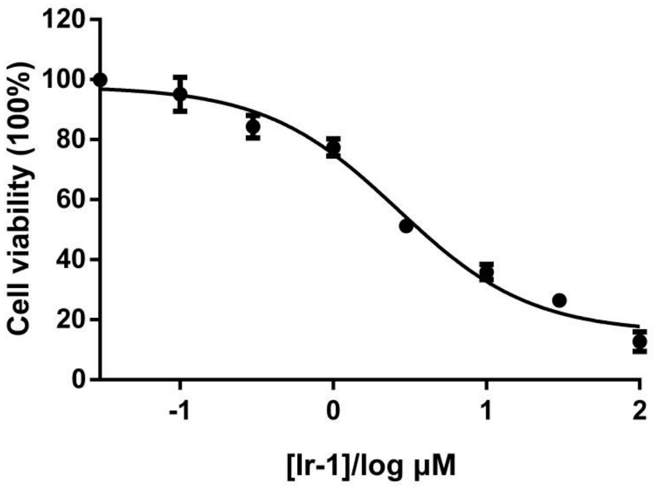

Fig. S9 The cytotoxicity effect of **Ir-1** towards HeLa cells as determined by an MTT assay. **Ir-1** inhibited the growth of HeLa cells with an IC_{50} value of 5.12 μM.

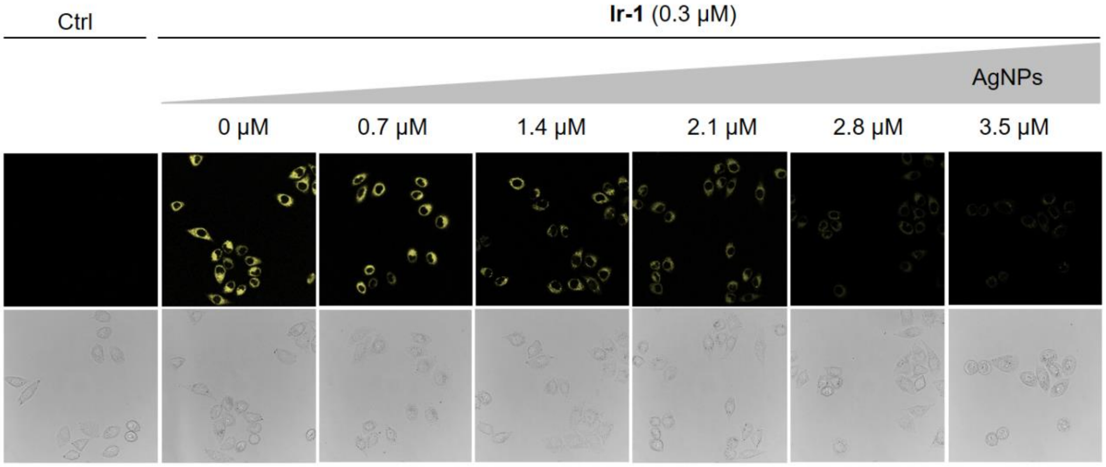

Fig. S10 Confocal luminescence microscopy imaging of HeLa cells which were incubated with **Ir-1** (0.3 μM) with increase of AgNPs from 0 to 3.5 μM.

Table S2. Comparison of optical probes for the detection of H_2O_2.

H_2O_2 sensors	Methods	Detection limit	Reference
Gold nanorods	Surface enhanced Raman spectroscopy	0.3 μM	4
Au@Ag nanoparticles	Electrocatalysis	1.3 μM	10
Silver nanoclusters	Fluorometric	0.4 μM	11
Mito-VH	Fluorometric	2.1 μM	13
Carbon dots	Fluorometric	0.5 μM	14
Co_3O_4-cored carbon dots	Chemiluminescence	10 μM	16
Polymeric nanoprobes	Fluorometric	0.95 μM	20
Ag nanoparticles-graphene nanocomposites	Surface enhanced Raman scattering	100 μM	39
Graphene oxide composites	Electrocatalysis	0.27 μM	42
Gold nanoparticles	Colorimetric	10 μM	43
Graphene–pt nanocomposites	Electrocatalysis	0.2 μM	44
Phenyl boronic acid-functionalized quinone-cyanine	Colorimetric and NIR Fluorescence	5.3 μM	45
SiO_2-coated graphene oxide	Electrocatalysis	4 μM	46
Iridium(III) complex-AgNps	Fluorometric	0.3 μM	This work

Reference

1. Crosby, G. A.; Demas, J. N., Measurement of photoluminescence quantum yields. Review. *J. Phys. Chem.* **1971,** *75* (8), 991-1024.
